# Supplementary material for: Comparative multiomics analysis of cell physiological state after culture in a basket bioreactor
Source: Sci Rep. 2022 Nov 23;12:20161. doi: 10.1038/s41598-022-24687-4 (PMC9686226; doi:10.1038/s41598-022-24687-4)
Supplement: Supplementary file 1 — Supplementary Information 1. [file 41598_2022_24687_MOESM1_ESM.zip › raw data/Transcriptomic raw data/7.AS/2.dasplot/TE1vsTC1/A3SS/19_BRD9_NW_023666054.1_35282431_35282543_-@NW_023666054.1_35282431_35282546_-@NW_023666054.1_35283193_35283303_-.pdf]

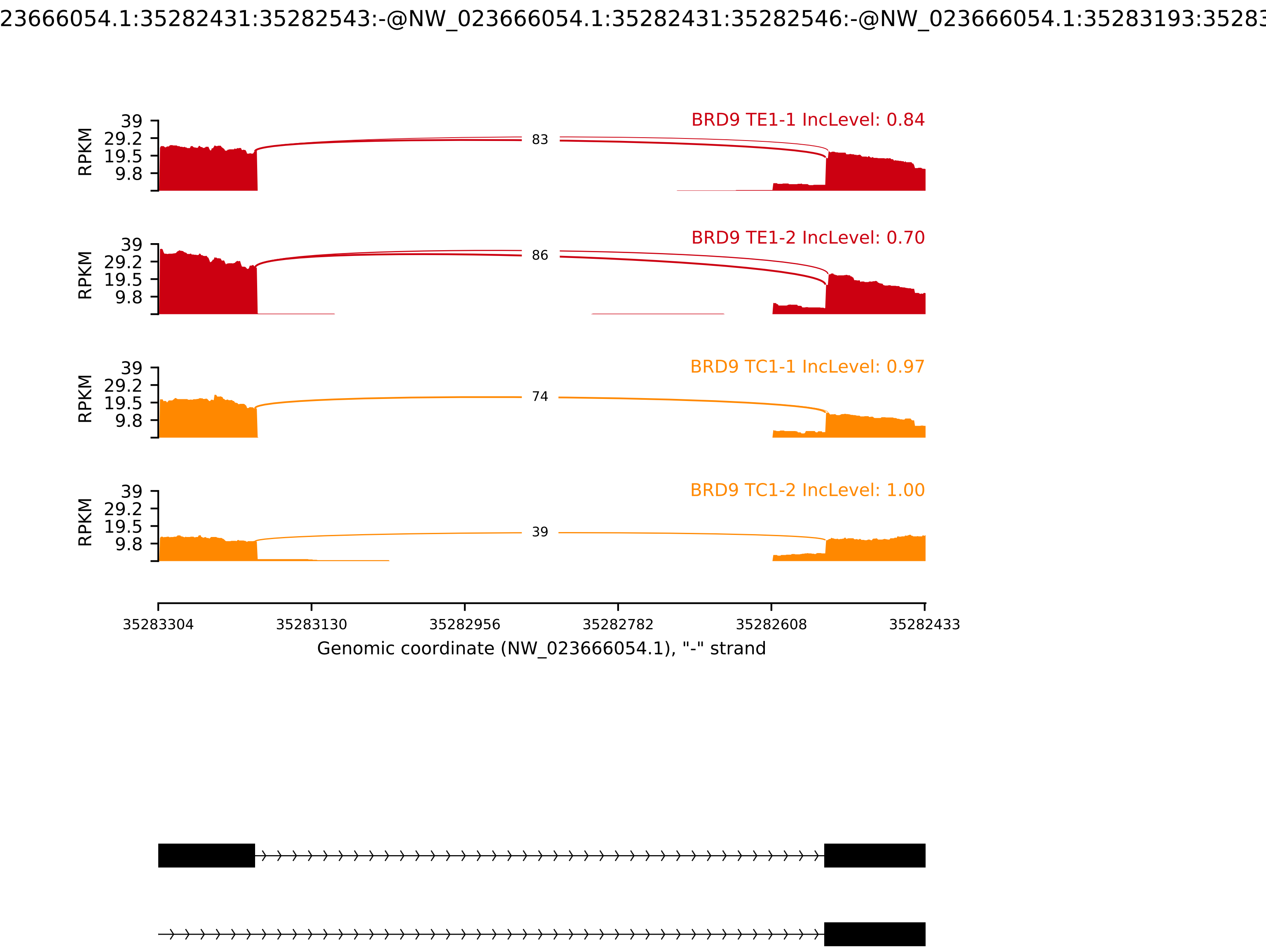
23666054.1:35282431:35282543:-@NW_023666054.1:35282431:35282546:-@NW_023666054.1:35283193:3528
BRD9 TE1-1 IncLevel: 0.84
83
BRD9 TE1-2 IncLevel: 0.70
86
BRD9 TC1-1 IncLevel: 0.97
74
BRD9 TC1-2 IncLevel: 1.00
39
RPKM
39
29.2
19.5
9.8
35283304
35283130
35282956
35282782
35282608
35282433
Genomic coordinate (NW_023666054.1), "-" strand
